# Molecular docking analysis of cetuximab with NOTCH signalling pathway targets for oral cancer

**DOI:** 10.6026/97320630019471

**Published:** 2023-04-30

**Authors:** Jayanthi Pazhani, Vishnu Priya Veeraraghavan, Selvaraj Jayaraman

**Affiliations:** 1 Centre of Molecular Medicine and Diagnostics (COMManD), Department of Biochemistry, Saveetha Dental College and Hospitals, Saveetha Institute of Medical and Technical Sciences, Saveetha University, Chennai-600077, India

**Keywords:** Oral carcinoma, NOTCH signaling, Cetuximab, molecular docking

## Abstract

Notch signaling is an evolutionarily ancient mechanism which intricated in cell-cell communication and it plays a crucial role in various developments in malignancies. Inactivating mutations of NOTCH targets are present in about 10 % of cases of squamous cell carcinoma of the skin, oral cavity, and esophagus that rendering it one of the most frequently mutated genes in oral squamous cell carcinoma. Therefore, it is of interest to document the molecular docking analysis of cetuximab with the NOTCH signaling targets such as NOTCH1, NICD, and HES1. These results suggest that targeting the NOTCH signaling with cetuximab might leads to the better outcome for suppression of invasion and metastasis in oral carcinoma.

## Background:

Oral cancer is the leading most common solid malignancies around worldwide, and also around 90% of oral cancers are mainly constitutes of oral squamous cell carcinoma (OSCC) [1]. At the advanced stages, OSCC are intense in invasive and metastatic that leads to the poor survival rate. Around the world, annually 145,000 mortalities in count and among 77% of cases are from the developing countries [2]. Anatomically, OSCC generally occurs in oral cavity, nasal, tongue, lips, saliva gland, and larynx [3]. The fundamental roles for the formation of OSCC are due to the environmental risk, viral infection, and genetic mutation [3]. For the nature of invasion and metastasis, OSCC has a deprived prognosis, and need a better improvements and innovations which could be cooperative in treatment [4]. There is a need for experimental improvements in oral cancer research [5]. Consequently, it is vital to study the basis molecular mechanism of OSCC that leads to the better development in therapeutic option for the devastating disease [6].

Cetuximab, a recombinant human-murine chimeric mAb, precisely binds to Epidermal Growth Factor Receptor (EGFR) on the cell surface and that hinders with the downstream signal transduction [7]. Consequently, the oral cancer drug, cetuximab inhibits tumor cell proliferation, invasion, metastasis, angiogenesis, cell cycle arrest, and promotes tumor cell apoptosis [8]. Moreover, cetuximab that can potentially induce immunologic anti-tumour effects, that so termed as antibody-dependent cell-mediated cytotoxicity (ADCC), since it mostly consists of a human IgG1 spine [9]. One of the previous studies reported that Phase III clinical trials have established that cetuximab have been used in combination with radiotherapy in oral squamous cell carcinoma (OSCC). [10] and Bonner *et al*., stated that the combination of cetuximab with platinum-based chemotherapy as the first-line treatment for OSCC that have attained a higher response rate and results momentously increase in Overall Survival rate [11]. In addition, cetuximab has been reported that cetuximab used in combination with paclitaxel is an effective way when platinum-based chemotherapy fails [12]. However, the efficacy of cetuximab for targeting NOTCH signaling remains poorly understood.

Notch signalling is an evolutionarily-conserved pathway that involved in cell death determination in various tissue types like squamous cells, epithelial, and mesenchymal cells during embryonic development [13]. Moreover, notch pathway targets which are actively affects a broad range of developmental processes, such as differentiation, proliferation, haematopoiesis, angiogenesis, growth/differentiation of keratinocytes, and craniofacial development, and also includes tooth development [14]. Molecular docking is a computational procedure that attempts to predict non-covalent binding of macromolecules or, more frequently, of a macromolecule (receptor) and a small molecule (ligand) efficiently, starting with their unbound structures, structures obtained from Molecular dynamic simulations, or homology modeling, *etc* [15]. Therefore, it is of interest to document the molecular docking analysis of cetuximab with the NOTCH signaling targets such as NOTCH1, NICD, and HES1.

## Materials and Methods

## Protein preparation:

The crystal structure of NOTCH signaling targets such as NOTCH1, NICD, and HES1 (NOTCH1 - PDB ID: 4D0F; NICD - PDB ID: 3V79; HES1 - PDB ID: 3NBN) was retrieved from Protein Data Bank for molecular docking studies. Prior to docking, protein was prepared using protein preparation wizard module that implemented in AutoDock. All the water molecules were deleted and hydrogen atoms were added to the structure. The orientation of amide, hydroxyl and thiol groups and the protonation, tautomeric state of these residues were optimized. Partial atomic charges were assigned according to the OPLS-2005 force field [16]. The structure was then subjected to impact minimization with a cut off RMSD of 0.3 Å.

## Ligand preparation:

The structure of Cetuximab was retrieved from pubchem. 32 stereoisomers were generated per ligand and all possible ionization of ligand was generated at pH of 7.0. Conformer generation of each ligand was carried out using Configuration generation module of AutoDock with default parameters. A maximum number of conformers were set as 1000 per ligand using both pre and post minimization steps that were set as 100 and 50 respectively. Each minimized conformer was filtered through a relative energy window of 10 Kcal mol-1 and a minimum atom deviation of 1.0 Å. This value (10 Kcal mol-1) sets an energy threshold relative to the lowest energy conformer. Conformers having higher energy than the threshold are discarded. Distances between all pairs of corresponding heavy atoms must be below 1.0 Å for two conformers to be considered identical. This criterion is applied only after energy difference threshold and only if two conformers are within 1 Kcal mol-1 [17].

## Molecular docking analysis:

Molecular docking analysis was performed using AutoDock 4.2 version. Prior to the docking, the binding site was predicted using SiteMap and grid was generated around the binding site using the receptor grid generation panel. The grid-enclosing box was cantered to the active sites of the corresponding 3D-structure of the receptor so as to enclose them within 3.0 Å from the centroid of amino acid residues. A scaling factor of 1.0 was set to van der Waals (vdW) radii of those receptor atoms with the partial atomic charge less than 0.25. Docking calculations were performed with XP mode, which performs systematic search of conformational, orientation and positional space of docked ligand, discarding unwanted conformations using scoring followed by energy optimization. The docking algorithm performs a series of hierarchical searches for the locations of possible ligand affinity within the binding site of NOTCH1, NICD, and HES1 with cetuximab complex.

##  Results and Discussion:

**Figure 1:**
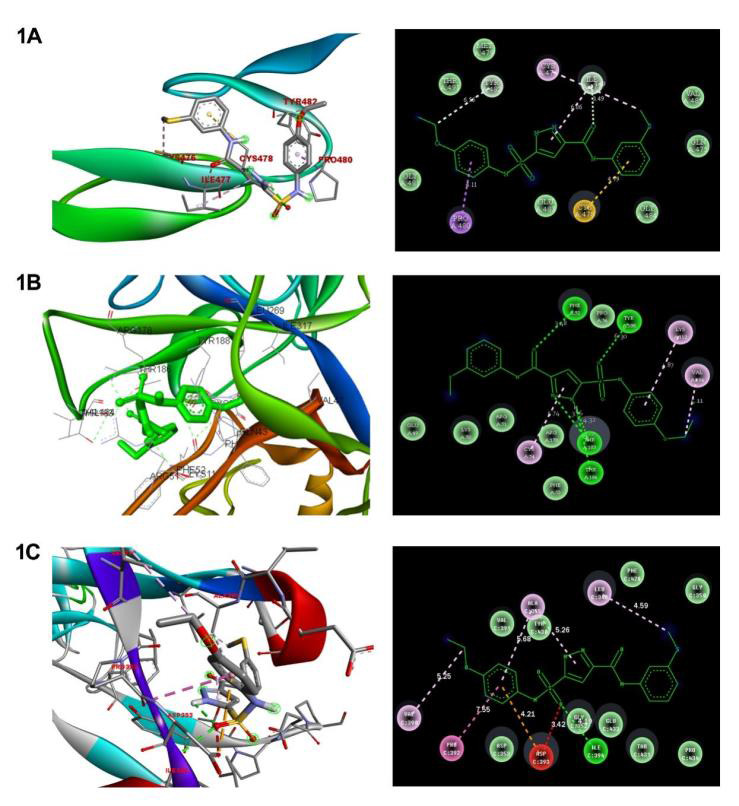
Molecular docking analysis of cetuximab with NOTCH signaling pathway targets such as NOTCH1, NICD and HES1

The determination of Prime position of a ligand to bind its target active site can be predicted using a Molecular Docking Tool. It is simulation techniques which will selects the three-dimensional coordinate space of the binding site in target and calculate the molecule's binding interaction within the binding site using the resultant orientation, which will form a complex. The sensitivity of binding affinity values can be calculated by the highest magnitude negative number, which indicates the most advantageous configuration of the complex. The Molecular Docking technique was performed with the drug Cetuximab in association with NOTCH signalling targets NOTCH1, NICD, HES1. The binding affinity of the drug Cetuximab against the target NOTCH1 measures -4.35(kcal/mol) which interacts with the TYR482, CYS478, PRO480 residues. The binding energy value of the drug against NICD target shows -6.16 (kcal/mol) interacting with THR186, GLN43 residues. The NOTCH signalling target HES1 has the binding affinity of -6.45(kcal/mol) when interacted with ALA355, ASP393 and PRO392 amino acid residues (Table 1 and Figure 1). Comparing within these targets the HES1 has the highest binding affinity energy against the drug Cetuximab. The 2D structure of the interactions was retrieved from Discovery Studio tool and the 3D structure of the interactions retrieved from Auto Dock Vina (ADT) ver1.5.4. This in-silico studies, shows that the Drug Cetuximab has the ability to inhibit the NOTCH signalling targets in the area of Oral cancer development. In-additional, the in-vitro and in-vivo studies can be helpful to analyse the overall process of the drug Cetuximab in oral cancer treatment.

## Conclusion:

We report the molecular docking analysis data for oral cancer drug, cetuximab with NOTCH signaling pathway targets such as NOTCH1, NICD and HES1 which regulates Epithelial Mesenchymal Transition (EMT). These results suggest significant results between the interaction of Cetuximab and NOTCH targets (NOTCH1, NICD, and HES1) which are mainly involved in Epithelial-mesenchymal Transition (EMT). Targeting the NOTCH signalling with cetuximab might be a better option for oral carcinoma.

## Figures and Tables

**Table 1:** Molecular docking results

**Drug**	**Proteins**	**Kcal/mol**	**Residues**
Cetuximab (CID-46507042)	NOTCH1(PDBID: 4DOF)	-4.35	TYR482,CYS478, PRO480
	NICD (PDB ID:3V79)	-6.16	THR186,GLN43
	HES1 (PDB:3NBN) ID:7X5E)	-6.45	ALA355,ASP393,PRO392 ARG72
